# Extract of *Triticum aestivum* Sprouts Suppresses Acetaminophen-Induced Hepatotoxicity in Mice by Inhibiting Oxidative Stress

**DOI:** 10.3390/molecules26216336

**Published:** 2021-10-20

**Authors:** Ji-Ye Lim, Dae-Ho Yun, Ji-Hyun Lee, Young-Bae Kwon, Young-Mi Lee, Dong-Hyun Lee, Dae-Ki Kim

**Affiliations:** 1 Department of Immunology, Institute of Medical Sciences, Medical School, Jeonbuk National University, Jeonju 54907, Korea; 84juce@naver.com (J.-Y.L.); jihyunsh1211@naver.com (J.-H.L.); 2 Department of Health Administration, Kwangju Women’s University, Kwangju 62396, Korea; ydh016@hanmail.net; 3 Department of Pharmacology, Institute for Medical Science, Medical School, Jeonbuk National University, Jeonju 54907, Korea; 1972y@jbnu.ac.kr; 4 Department of Oriental Pharmacy, College of Pharmacy and Wonkwang-Oriental Medicines Research Institute, Wonkwang University, Iksan 54538, Korea; ymlee@wku.ac.kr; 5 Department of Obstetrics and Gynecology, Jeonbuk National University Hospital, Jeonju 54907, Korea

**Keywords:** acetaminophen, apoptosis, hepatotoxicity, oxidative stress, *Triticum aestivum* sprouts

## Abstract

Wheat (*Triticum aestivum* L.) is the oldest known food crop, and many studies have reported that wheat shoots (i.e., wheatgrass) possess anti-cancer, anti-inflammatory, and antioxidant activities. However, the potentially ameliorative effect of wheat shoots on hepatotoxicity caused by high doses of *N*-acetyl-para-aminophenol (acetaminophen, APAP) has yet to be reported. C57BL/6 mice received daily oral TAE (100 or 200 mg/kg), positive control (silymarin 100 mg/kg), or negative control (saline vehicle) treatments for 7 days prior to intraperitoneal APAP injection. Histological, serum (ELISA), Western blotting, and quantitative PCR analyses of excised liver tissues were then performed. Pre-treatment with TAE (100 or 200 mg/kg) ameliorated APAP-induced pathological damage (i.e., hepatotoxic lesions), reduced serum alanine aminotransferase (ALT) and aspartate aminotransferase (AST) levels, and also ameliorated APAP-induced increases in oxidative stress, thereby inhibiting oxidative liver damage and reducing the expression of inflammatory cytokines. In addition, TAE pre-treatment inhibited the expression of Cytochrome P4502E1 (CYP2E1), which is a key enzyme in the onset of APAP-induced hepatotoxicity, suppressed the expression of the target proteins regulated by the antioxidant enzyme Nrf2, and suppressed hepatocyte apoptosis. These findings suggest that TAE is an attractive therapeutic candidate that exhibits potential hepatoprotective activity by inhibiting oxidative stress, inflammation, apoptosis, and liver damage.

## 1. Introduction

Acetaminophen (*N*-acetyl-para-aminophenol (APAP)) is a widely used pain reliever and antipyretic and is considered safe at therapeutic doses [1]. However, as many people take it, drug addiction is common, and many studies have reported that death can occur as a result of liver damage and acute liver failure [1]. After ingestion, most APAP (85%) is conjugated with sulfuric acid or glucuronic acid and excreted in urine without hepatotoxicity. However, 4% is oxidized by cytochrome P450 (CYP450) into *N*-acetyl-p-benzoquinone imine (NAPQI), an intermediate metabolite [1]. The transient receptor potential ankyrin-1 (TRPA1) channel is expressed by peptidergic primary sensory neurons [2]. NAPQI is a powerful TRPA1 agonist, and the analgesic effect of APAP causes the action of metabolites of the parent drug on sensory neuron TRP channels, preventing nerve cells from transmitting information and thereby attenuating the transmission of pain signals to the brain [3].

NAPQIs are highly toxic substances that directly damage mitochondria [4] and cause the induction of inflammatory responses, and play the most important role in initiating apoptosis [5], which results in the formation of reactive oxygen species (ROS) inside mitochondria, and, thus, impairs mitochondrial function [4]. The intentional or unintentional overuse of APAP can cause severe liver damage and acute liver failure in both humans and laboratory animals. In addition, most APAPs are metabolized by cytochrome P450 in the liver, and NAPQI production increases, exhausting glutathione (GSH) in the liver cells, causing severe liver cell death due to cytotoxicity [1]. This results in a secondary activation of the innate immune response associated with the upregulation of inflammatory cytokines and the activation of natural killer (NK) cells, NKT cells, and neutrophils [6]. Since the major pathological change in liver damage caused by APAP is oxidative stress, it is important to discover antioxidants that are effective in alleviating hepatotoxicity [7].

Wheat (*Triticum aestivum* L., (TA), which is the oldest known food crop, remains a major crop global crop and is an excellent source of biologically active substances [8]. TA shoots (i.e., wheatgrass) are richer than mature plants, witha variety of nutrients, vitamins, minerals, and proteins [8]. Many studies have also reported that TA possesses anti-cancer [9], anti-inflammatory [10], and antioxidant [11] properties. TA is effective for treating several diseases, including atopic dermatitis-like skin lesions [8], alcoholic liver damage [12], LPS-induced liver injury [13], and allergies [14]. However, no previous studies have investigated the hepatoprotective effects and mechanisms of TA on the toxic effects of drug, such as APAP. Due to the nature of liver function, the risk of liver disease can be lowered by eating foods that prevent liver toxicity. Therefore, the goals of the present study were to evaluate the effect of TA on APAP-induced hepatotoxicity in mice and to elucidate the in vivo antioxidant signaling mechanisms that mediate this effect.

## 2. Results

### 2.1. Chemical Properties of TAE

**Figure 1:**
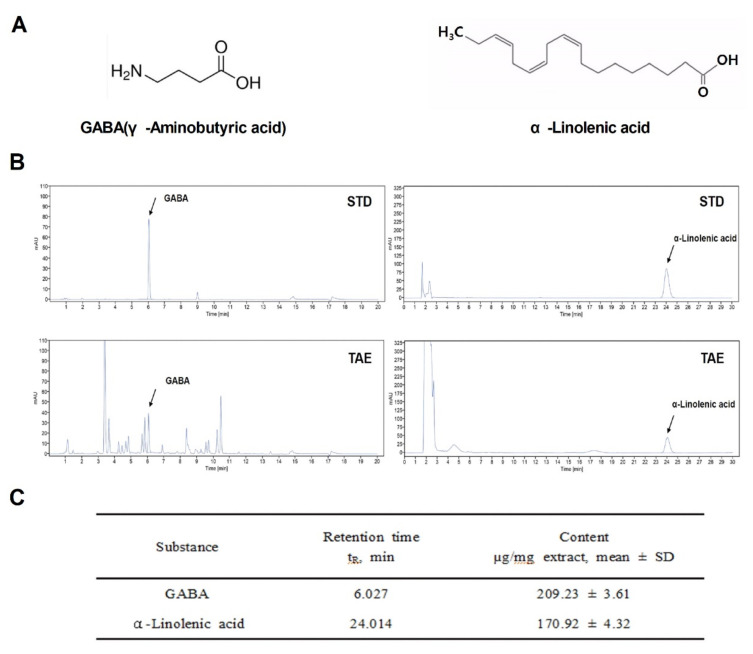
Chemical components of an ethanolic extract of *Triticum aestivum* sprouts. (**A**) Chemical structures of the two identified components (GABA and α-Linolenic acid). (**B**) Liquid chromatograms of standard compound mixtures (STD) and *Triticum aestivum* sprouts extract (TAE). (**C**) Quantification of isolated compounds from chromatograms (mean ± SD, *n* = 3). TAE, *Triticum aestivum* sprouts extract.

During germination, wheatgrass contains a variety of active ingredients: amino acids, minerals, vitamins and chlorophyll. Among them, GABA [15], a representative indicator substance, and α-Linolenic acid were analyzed based on the results of previous studies [16]. To study the potential regulatory role of TAE as a therapeutic agent in APAP-induced liver damage, the chemical structures of indicator compounds (GABA and α-linolenic acid) were identified and analyzed using HPLC and UPLC (Figure 1A,B). The final extraction yield (%) of the TAEs was 25%. Two compounds in the TAE extracts were identified (Figure 1B), namely GABA and α-linolenic acid, which were also quantified (Figure 1C). It was confirmed that the extract was detected at the same retention time as the indicator compound.

### 2.2. Effect of TAE on APAP-Induced Hepatotoxicity 

**Figure 2:**
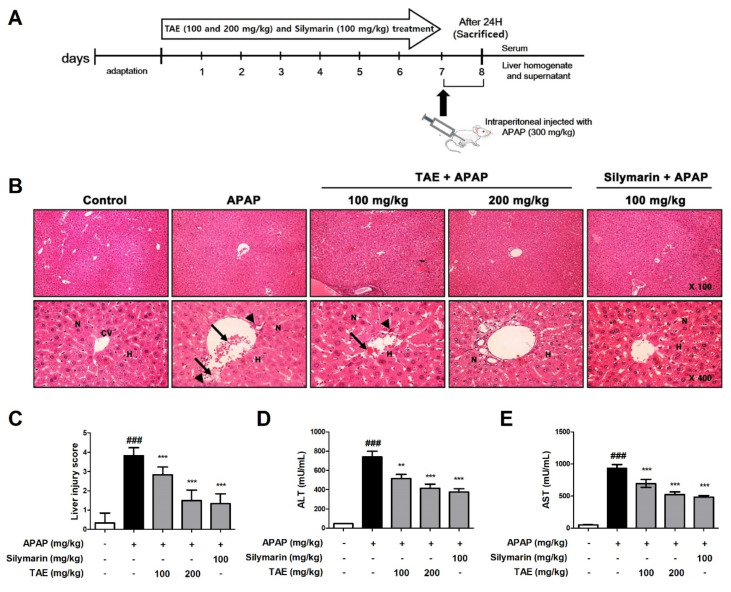
Effect of ethanolic *Triticum aestivum* sprout extract on *N*-acetyl-para-aminophenol (APAP)-induced hepatotoxicity lesions in mice. (**A**) Experimental scheme. (**B**) Representative images of Hematoxylin and eosin (H&E)-stained liver sections from mice (×100 and ×400 magnification); hepatic architnormalecture with central vein (CV), surrounding hepatocytes (H), nucleus (N), hepatic centrilobular mononuclear cell infiltration (long arrow) and hepatic cell necrosis (arrowhead). (**C**) Liver tissue damage score. (**D**) Serum levels of alanine aminotranserfase (ALT) activity, as indicated by ELISA. (**E**) Serum levels of aspartate aminotransferase (AST) activity, as indicated by ELISA. All data are shown as mean ± SD. ### *p* < 0.001 versus Normal group; ** *p* < 0.01, and *** *p* < 0.001 versus APAP group. ALT, alanine aminotransferase; APAP, *N*-acetyl-para-aminophenol (acetaminophen); AST, aspartate aminotransferase; TAE, *Triticum aestivum* sprouts extract.

The histological examination revealed that APAP induced the destruction of the liver structure around blood vessels, hepatic mesenchymal necrosis, and the infiltration of inflammatory cells (Figure 2). However, pre-treatment with TAE (100 or 200 mg/kg) attenuated the formation of liver tissue lesions in a dose-dependent manner, and the TA group that received the higher TAE pre-treatment TAE (200 mg/kg) was similar in terms of structural improvement to that of the positive control (silymarin 100 mg/kg) (Figure 2B,C).

Next, we measured and confirmed changes in ALT and AST levels in the serum of mice with APAP-induced hepatotoxicity (Figure 2D,E). APAP increased serum ALT and AST levels, and TAE pre-treatment reduced these increases.

### 2.3. Effect of TAE on APAP-Induced Liver Oxidative Stress

**Figure 3:**
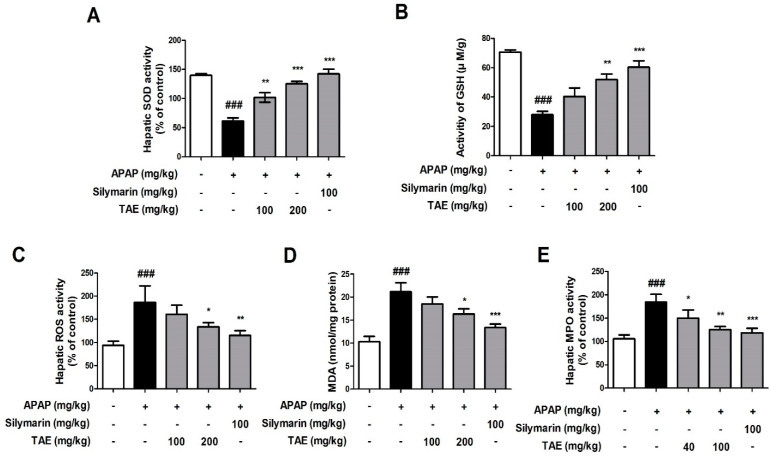
Effect of ethanolic *Triticum aestivum* sprout extract on *N*-acetyl-para-aminophenol (APAP)-induced liver oxidative stress in mice livers. (**A**) SOD activity. (**B**) GSH activity. (**C**) ROS activity. (**D**) MDA activity. (**E**) MPO activity. All data are shown as mean ± SD. ### *p* < 0.001 versus Normal group; * *p* < 0.05, ** *p* < 0.01, and *** *p* < 0.001 versus APAP group. APAP, *N*-acetyl-para-aminophenol (acetaminophen); GSH, glutathione; MDA, malondialdehyde; MPO, myeloperoxidase; ROS, reactive oxygen species; SOD, superoxide dismutase; TAE, *Triticum aestivum* sprouts extract.

Changes in SOD, GSH, ROS, MDA, and MPO activity levels indicated that APAP induced oxidative liver damage (Figure 3). More specifically, both SOD and GSH activities were significantly reduced by APAP treatment, whereas ROS, MDA, and MPO activities were elevated, and TAE pre-treatment (100 or 200 mg/kg) ameliorated these changes, which indicates that TAE can inhibit oxidative liver damage in APAP-induced hepatotoxicity.

### 2.4. Effect of TAE on APAP-Induced Inflammatory Cytokine 

**Figure 4:**
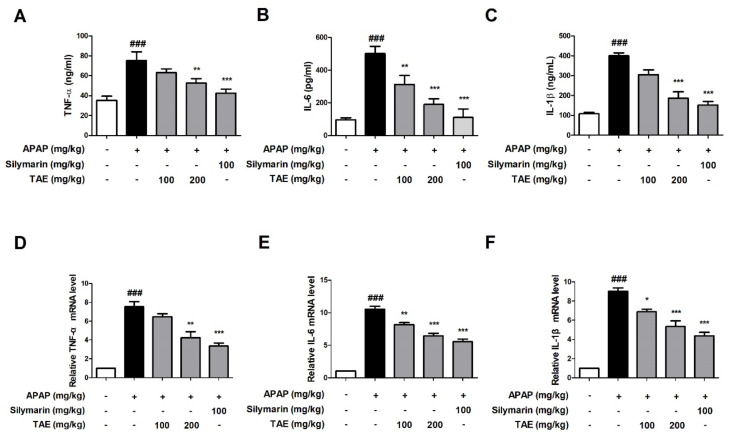
Effect of ethanolic *Triticum aestivum* sprout extract on inflammatory cytokine production in mice with *N*-acetyl-para-aminophenol (APAP)-induced hepatotoxicity. Inflammatory cytokine levels were confirmed by ELISA and qRT-PCR. (**A**) TNF-α expression. (**B**) IL-6 expression. (**C**) IL-1β expression. (**D**) TNF-α mRNA expression. (**E**) IL-6 mRNA expression. (**F**) IL-1β mRNA expression. All data are shown as mean ± SD. ### *p* < 0.001 versus Normal group; * *p* < 0.05, ** *p* < 0.01, and *** *p* < 0.001 versus APAP group. APAP, *N*-acetyl-para-aminophenol (acetaminophen); GAPDH, glyceraldehyde 3 phosphate dehydrogenase; IL, interleukin; TNF-α, tumor necrosis factor-alpha; qPCR: Quantitative polymerase chain reaction; TAE, *Triticum aestivum* sprouts extract.

We compared the results to the control group, confirming that the APAP-induced group (*p* < 0.01) significantly increased the serum and mRNA levels of TNF-α, IL-6, and IL-1β. The present study evaluated whether TAE could inhibit the accumulation of inflammatory cytokines at both the serum and mRNA levels. The serum levels of all three cytokines were significantly elevated in the APAP group when compared to the control group (*p* < 0.01), whereas the pre-treatment with TAE (100 or 200 mg/kg) reduced cytokine levels, with an efficacy similar to that observed in the positive control group (Figure 4A–C). mRNA analysis also indicated that TAE pre-treatment inhibited the upregulation of inflammatory genes in the liver tissues of mice experiencing APAP-induced hepatotoxicity (Figure 4D–F).

### 2.5. Effect of TAE on CYP2E1 and Nrf2 Pathway during APAP-Induced Hepatotoxicity

**Figure 5:**
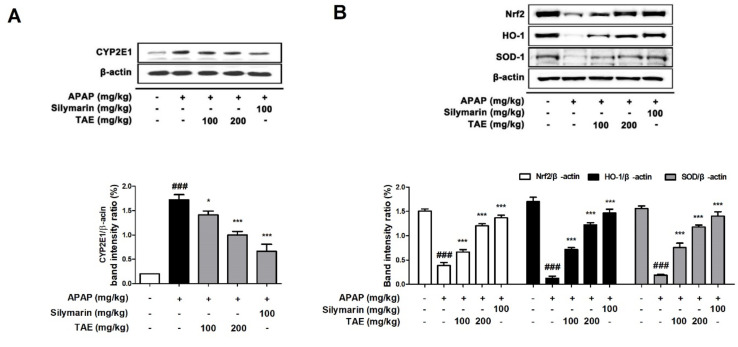
Effects of ethanolic *Triticum aestivum* sprout extract on CYP2E1 and Nrf2 pathway proteins in mice with *N*-acetyl-para-aminophenol (APAP)-induced hepatotoxicity. (**A**) Quantitative analysis of CYP2E1 protein expression. (**B**) Quantitative expression analysis of Nrf2 regulatory target proteins: Nrf2//β-actin, HO-1/β-actin, and SOD-1/β-actin. All data are shown as mean ± SD. ### *p* < 0.001 versus Normal group; * *p* < 0.05, ** *p* < 0.01, and *** *p* < 0.001 versus APAP group. APAP, *N*-acetyl-para-aminophenol (acetaminophen); CYP2E1, Cytochrome P4502E1; HO-1, heme oxygenase-1; Nrf2, Nuclear factor erythroid **2**-related factor 2; SOD, superoxide dismutase; TAE, *Triticum aestivum* sprouts extract.

Cytochrome P4502E1 (CYP2E1) is a key enzyme that explains the metabolism of APAP to the toxic substance NAPQI [1]. Therefore, a Western blot was used to investigate whether TAE affects the protein expression of CYP2E1 in mice liver. CYP2E1 was significantly upregulated in the APAP group (*p* < 0.05). However, pre-treatment with TAE (100 or 200 mg/kg) significantly inhibited CYP2E1 expression in a dose-dependent manner, with an efficacy similar to that observed in the positive control group (Figure 5A). Nuclear factor erythroid **2**-related factor 2 (Nrf2) is a representative mechanism involved in antioxidant activity in the body and is a transcription factor that plays an important role in the activation of cellular antioxidant enzymes against oxidative stress [17]. It is also recognized as a potential therapeutic target for chemical-induced liver damage [18]. We confirmed the levels of Nrf2-regulated target proteins such as heme oxygenase-1 (HO-1) and SOD (Figure 5B). Nrf2 was downregulated in the APAP group (*p* < 0.05), as expected, and TAE pre-treatment (100 or 200 mg/kg) ameliorated the suppression of Nrf2, HO-1, and SOD-1 in a dose-dependent manner.

### 2.6. Effect of TAE on ASK1 and JNK Phosphorylation

**Figure 6:**
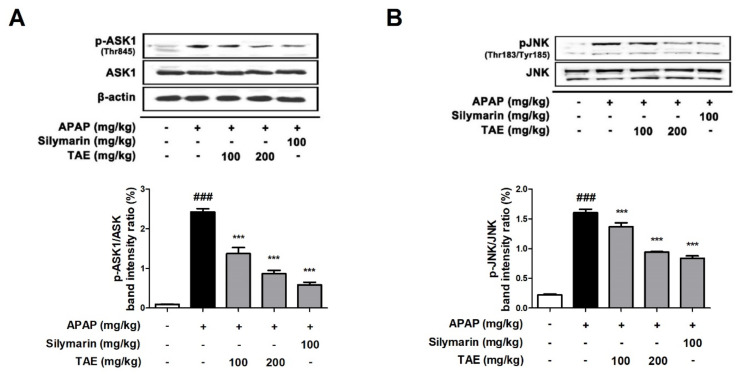
Effect of ethanolic *Triticum aestivum* sprout extract on JNK phosphorylation in mice with *N*-acetyl-para-aminophenol (APAP)-induced hepatotoxicity. (**A**) Quantitative analysis of phosphorylated ASK1 protein. (**B**) Quantitative analysis of phosphorylated JNK protein. The bar graph represents the quantitative band densities of pASK1/ASK1 and pJNK/JNK. All data are shown as mean ± SD. ### *p* < 0.001 versus Normal group; *** *p* < 0.001 versus APAP group. ASK1, apoptosis signaling regulatory kinase 1; APAP, *N*-acetyl-para-aminophenol (acetaminophen); JNK, c-jun *N*-terminal protein kinase cascade; TAE, *Triticum aestivum* sprouts extract.

NAPQI generated by an overdose of APAP increases ROS production, causing the phosphorylation of JNK and can further amplify oxidative stress [4,18]. Additionally, it has been reported that apoptosis signaling regulatory kinase 1 (ASK1) was identified in the c-jun *N*-terminal protein kinase (JNK) cascade during APAP-induced hepatotoxicity [19]. The Western blot analysis revealed that TAE pre-treatment (100 or 200 mg/kg) suppresses the phosphorylation of both ASK and JNK in a dose-dependent manner (Figure 6), with an efficacy similar to that observed in the positive control group.

### 2.7. Effect of TAE on Hepatocyte Apoptosis in APAP-Induced Hepatotoxicity

**Figure 7:**
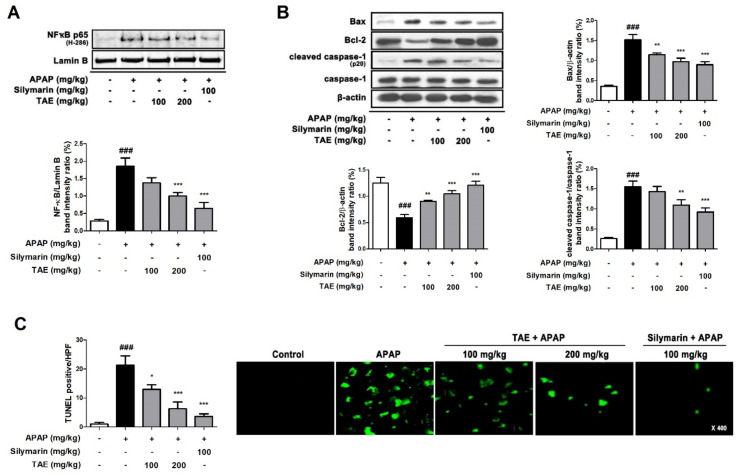
Effect of ethanolic *Triticum aestivum* sprout extract on hepatocyte apoptosis in mice with *N*-acetyl-para-aminophenol (APAP)-induced hepatotoxicity. (**A**) Quantification of nuclear translocation of the p65 subunit of NF-κB. (**B**) Quantification Bax, Bcl-2, cleaved caspase-1, and caspase-1 protein expression. (**C**) Quantification and visualization (×400 magnification) of DNA fragmentation. All data are shown as mean ± SD. ### *p* < 0.001 versus Normal group; * *p* < 0.05, ** *p* < 0.01, and *** *p* < 0.001 versus APAP group. APAP, *N*-acetyl-para-aminophenol (acetaminophen); Bax, Bcl-2-associated X; Bcl2, B-cell lymphoma 2; Caspase, cysteinyl aspartate specific proteinase; NF-κB, transcription factors nuclear factor-kappa B; TAE, *Triticum aestivum* sprouts extract.

APAP-induced hepatotoxicity significantly increased the nuclear transcription of NF-κB (p65) and Bcl-2-associated X (Bax) and induced the cleavage of cysteinyl aspartate specific proteinase (caspase)-1, a marker of inflammatory activation (Figure 7A,B). However, the pre-treatment of TAE (100 or 200 mg/kg) and positive control (silymarin 100 mg/kg) significantly reduced this elevation. The TUNEL assay showed that the apparent TUNEL-positive cells were detected by fluorescence in the APAP group, whereas TAE (100 or 200 mg/kg) pre-treatment significantly reduced the number of TUNEL-positive cells in a dose-dependent manner. In particular, the high TAE (200 mg/kg) pre-treatment significantly reduced the increased fluorescence intensity, similar to the positive control (silymarin 100 mg/kg) (Figure 7C).

## 3. Discussion

Acetaminophen (APAP) is widely used to cause acute oxidative liver damage in research models [20]. The compound is generally considered safe, since it is detoxified and excreted by antioxidant defense mechanisms when taken at appropriate concentrations, and is the main component of a variety of antipyretic and analgesic drugs, such as Tylenol [1]. However, overdoses of APAP result in the depletion of GSH, which detoxifies the active metabolite NAPQI, and this results in oxidative damage to cell membranes and intracellular macromolecules, thereby damaging liver cells [4,21]. As a result, extensive research has been conducted to isolate hepatoprotective compounds from traditional herbal medicines and natural compounds with various pharmacological mechanisms and fewer side effects [22].

The present study evaluated the hepatoprotective effects of TAE in mice with APAP-induced hepatotoxicity. Overdoses of APAP result in the release of large amounts of ALT and AST, which significantly increases blood volume and, thus, can seriously damage hepatocytes [18]. However, the histopathological results of the present study indicate that TAE pre-treatment reduced nuclear loss and damage to liver structural morphology, and TAE pre-treatment also alleviated the APAP-induced elevation of ALT and AST activity. These findings suggest that TAE pre-treatment can reduce hepatocellular damage during APAP overdose by inhibiting the release of ALT and AST from the serum.

The production of ROS and prevention of lipid peroxidation are the most common mechanisms by which natural compounds provide liver protection [18]. Moreover, MDA is the end-product of lipid peroxidation and indirectly reflects the production of ROS in organisms [18]. In such ROS-rich environments, MPO, which is an indicator of oxidative damage, increases in proportion to the degree of penetration by neutrophils, thereby generating reactive radicals and causing oxidative stress that exacerbates cellular damage [13,18]. In the present study, TAE significantly upregulated the expression of SOD and GSH and downregulated ROS, MDA, and MPO levels in a dose-dependent manner, which suggests that TAE can potently inhibit APAP-induced oxidative liver damage.

Metabolic activation by APAP induces inflammatory cell infiltration and the overexpression of inflammatory cytokines, such as TNF-α, IL-6, and IL-1β, ultimately leading to inflammation [18]. In the present study, APAP injection significantly upregulated both the serum and mRNA levels of TNF-α, IL-6, and IL-1β, whereas TAE pre-treatment down-regulated them. Thus, the hepatoprotective effects of TAE in this APAP-induced hepatotoxicity model are also associated with anti-inflammatory activity.

Oxidative stress plays an important role during APAP-induced hepatotoxicity [13,18]. Since the NAPQI toxins produced by APAP are metabolized by the CYP pathway, and especially by CYP2E1, the antioxidant properties of TAE in APAP-induced hepatotoxicity may be partially related to the inhibition of CYP enzymes [18,23]. Furthermore, because CYP2E1 is a major contributor to APAP-induced hepatotoxicity, the inhibition of CYP2E1 may be a promising therapeutic strategy for addressing APAP-induced hepatotoxicity. In our study, APAP injection significantly increased CYP2E1 expression, whereas TAE pre-treatment significantly suppressed CYP2E1 upregulation in a dose-dependent manner. Thus, we suggest that the hepatoprotective action of TAE is also mediated by the removal of NAPQI toxins. It has been demonstrated that the activation of Nrf2 by pharmacologically active agents or genetic manipulation plays an important role in protecting the liver from APAP-induced hepatotoxicity in mice and in alleviating chemically induced oxidative stress damage [17]. Thus, the Nrf2-regulated antioxidant system plays a key role in mitigating chemically induced oxidative stress damage [17,18]. Interestingly, TAE pretreatment confirmed that the protein levels of Nrf2, HO-1, and SOD-1 were significantly reduced by APAP injection, and particularly significantly inhibited these reductions in HO-1 and SOD-1.

The upstream kinase of JNK activation is activated by ASK1 in APAP-induced liver injury [19]. In the present study, TAE pre-treatment inhibited the APAP-induced phosphorylation of ASK1 and JNK. Therefore, TAE exhibited hepatoprotective activity through the inhibition of ASK1 phosphorylation during APAP-induced hepatotoxicity and suppression of oxidative stress by downregulating JNK phosphorylation.

The APAP-induced upregulation of Bax is attenuated by the inhibition of JNK activation [19,23,24]. APAP has been reported to induce and inhibit the expression of Bax (pro-apoptotic factor) and Bcl-2 (anti-apoptotic factor), respectively, and the upregulation of the Bax/Bcl-2 ratio damages the mitochondrial membrane and induces a caspase-dependent apoptosis pathway [24,25], with resulting hepatocyte death that could promote the onset and progression of liver disease [25]. The findings of the present study indicate that APAP overdose induces typical signs of apoptosis, including caspase 1 activation and DNA fragmentation. However, TAE pre-treatment suppresses these signs, which clearly indicates that it contains an active ingredient with therapeutic potential for hepatotoxicity.

## 4. Materials and Methods

### 4.1. Chemicals and Reagents

The APAP, silymarin, chloroform and isopropanol used in the present study were purchased from Sigma-Aldrich (St. Louis, MO, USA). Cytokine measurement ELISA kits were purchased from BioLegend (San Diego, CA, USA). Specific antibodies used for Western blotting were purchased from Santa Cruz Biotechnology (Santa Cruz, CA, USA) and Abcam (Cambridge, UK). Commercial kits for measuring serum levels of alanine aminotransferase (ALT), aspartate aminotransferase (AST), reactive oxygen species (ROS), superoxide dismutase (SOD), glutathione (GSH), myeloperoxidase (MPO) activity, and malondialdehyde (MDA) were purchased from BioVision (Milpitas, CA, USA).

### 4.2. Ethanolic Extraction 

Wheat sprouts were obtained from the National Institute of Crop Science (Jeonbuk, Korea) and freeze-dried. Seeds were germinated on sterilized organic peat moss, and TA shoot extraction was performed as described previously [8]. Briefly, the shoots were powdered, and 30 g portions were ultrasonically extracted in 30% ethanol and filtered using Whatman filter paper (grade no. 1, diameter: 15 cm). These were stored at −80 °C until further analysis.

### 4.3. Chemical Analysis

The γ-Aminobutyric acid (GABA) contents of the TAEs, were measured using an OPA/FMOC derivatization reagent in high-performance liquid chromatography (HPLC; Agilent Technologies, Santa Clara, CA, USA), according to the manufacturer’s protocol. Meanwhile, the α-linolenic acid contents were measured using ultra-performance liquid chromatography (UPLC) on a Waters Acquity system (Waters, Milford, MA, USA). The UPLC was performed using 0.1% H_3_PO_4_ (pH 2.87) and, CH_3_CN as solvents (A and B, respectively), a mobile phase flow rate of 0.6 mL/min, detection at UV 206 nm, and temperature of 30 °C.

### 4.4. Experimental Design and Animals

Male C57BL/6 mice (6 weeks, 20–23 g, *n* = 35) were purchased from Samtako Bio Korea (South Korea). All mice were provided with adequate food and water and were housed in an animal room under standard conditions (21–24 °C, humidity, 45–60%, 12 h photoperiod). Mice were divided into 5 groups (mice/group). During a 7-day pre-treatment period, a TA group and positive control group were orally administered TAE (100 or 200 mg/kg) and silymarin (100 mg/kg) [26], respectively, whereas the vehicle group (control group) and APAP group received only phosphate-buffered saline (PBS). The TAE concentrations used in the experiment were based on data from previous studies [13]. Then, after the 7-day pre-treatment and 15 h of fasting, all individuals, except those in the control group, received intraperitoneal (i.p.) injections of APAP (300 mg/kg) [6]. Finally, at 24 h after injection, the mice were euthanized, and blood and liver samples were collected (Figure 2A). All animal procedures were approved by the Jeonbuk National University Laboratory Animal Care and Use Committee and were performed according to the Experimenta Ethics Committee (JBNU 2021-022).

### 4.5. Histological Observation

To evaluate the degree of APAP-induced hepatotoxicity, liver tissue samples were fixed in 10 % formalin and then embedded in paraffin wax. In order to evaluate the degree of liver tissue damage and nuclear DNA fragmentation, tissue samples embedded in paraffin were cut to a thickness of 5 μm and attached to a glass slide. After staining with hematoxylin-eosin (H&E), the degree of damage to the liver tissue was observed under an optical microscope (Olympus, Tokyo, Japan). Histopathological alterations were evaluated by randomly selecting 4 tissue samples per group. The liver tissue damage score was scored on a scale of of 0–4 and was evaluated as previously described [27].

### 4.6. ALT and AST Assays

Blood samples were obtained from a vein, and the serum was separated by centrifugation at 3500× *g* for 10 min. To evaluate liver function, serum alanine aminotransferase (ALT) and aspartate aminotransferase (AST) activities were measured using commercial diagnostic assay kits.

### 4.7. Cytokine Analysis

Levels of tumor necrosis factor-α (TNF-α), interleukin-6 (IL-6), and interleukin-1β (IL-1β) were commercially available ELISA kits, according to the manufacturer’s instructions.

### 4.8. Gene Expression Analysis

RNA was treated with Tirol reagent (Invitrogen, Carlsbad, CA, USA) according to the manufacturer’s instructions to dissolve the cell membrane, added to chloroform, and centrifuged to separate layers. After that, the aqueous phase was picked and transferred to a new tube, and isopropanol was added to extract only RNA, followed by centrifugation. Then, RNA pellet was used in 75 % ethanol to remove Na+ attached to the RNA. Total RNA was reverse transcribed into cDNA using PrimeScriptTM II 1st Strand cDNA synthesis kit (Promega, Madison, WI, USA). The qRT-PCR was performed using a SYBR Green Master Mix (Thermo Fisher Scientific Inc., Waltham, MA, USA) on StepOne Real-Time PCR System (Applied Biosystems). Mouse primers of inflammatory cytokines are described in Table 1, and all gene expression values were normalized according to the expression of glyceraldehyde 3-phosphate dehydrogenase (GAPDH) [27].

### 4.9. Biochemical Analysis 

Fresh liver tissue from each specimen was washed in cold PBS and stored at −80 °C for further analysis. Levels of myeloperoxidase (MPO), malondialdehyde (MDA), superoxide dismutase (SOD), and glutathione (GSH) activity were later analyzed using a commercially available kit according to the manufacturer’s protocol.

### 4.10. Protein Expression Analysis

Mice liver tissue was washed with fresh PBS and homogenized using radioimmunoprecipitation assay (RIPA) buffer. Protein was quantified using the BCA protein assay kit, and Western blotting was performed as previously [27]. Briefly, proteins were separated using 10% sodium dodecyl sulfate-polyacrylamide gel electrophoresis (SDS-PAGE), transferred to a polyvinylidene fluoride (PVDF) membrane (Millipore, USA) and quantified using specific antibodies and an enhanced chemiluminescent developing reagent (Advansta, San Jose, CA, USA).

### 4.11. DNA Fragmentation Analysis

DNA fragmentation was assessed using a terminal deoxynucleotidyl transferase dUTP nick end labeling (TUNEL) assay [27]. Tissue section slides were evaluated according to the manufacturer’s instructions and analyzed using a confocal microscope (Axio Vert.A1; ZEISS, Dublin, CA, USA).

### 4.12. Statistical Analysis

All experimental results are expressed as mean ± S.D. Statistical analyses were performed using GraphPad Prism (v.5.0) and one-way analysis of variance (ANOVA) was used to identify differences among the groups. The Bonferroni post hoc test was used for post-test comparison to the control and treatment groups. Statistical significance was set at *p* < 0.05.

## 5. Conclusions

The present study provides evidence for the hepatoprotective effects of pre-treatment with an ethanolic *Triticum aestivum* sprout extract in hepatotoxic environments. In summary, TAE pre-treatment was demonstrated to inhibit oxidative stress, inflammation, apoptosis, and liver damage during APAP-induced hepatotoxicity. Although the effects of TAE on other liver diseases remain unknown, the present study provides evidence that supports the use of traditional herbal medicines and natural compounds that may have fewer side effects than conventional drugs. The study indicates that TAE pre-treatment is an attractive candidate therapy for the prevention of hepatotoxicity. Meanwhile, the specific molecular mechanisms and precise targets of TAE are still unknown, and further studies are needed.

## Figures and Tables

**Table 1:** Primer sequences for qPCR.

Gene	Base sequence (5′-3′)	Size (bp)
mTNF-α-F	TAGCCAGGAGGGAGAACAGA	127
mTNF-α-R	TTTTCTGGAGGGAGATATGG	
mIL-6-F	GACAACCACGGCCTTCCCTA	302
mIL-6-R	GGTACTCCAGAAGACCAGAGGA	
mIL-1β-F	GCAACTGTTCCTGAACTCAACT	89
mIL-1β-R	ATCTTTTGGGGTCCGTCAACT	
mGAPDH-F	GAAGGTGAAGGTCGGAGT	226
mGAPDH-R	GAAGATGGTGATGGGATTTC	

TNF-α, tumornecrosis factor-α; IL-, Interleukin-; GAPDH, glyceraldehyde 3-phosphate dehydrogenase.

## Data Availability

The data presented in this study are available on request from the corresponding author.

## Sample Availability

Samples of the compounds are available from the authors.

## Abbreviations

ALT	Alanine aminotransferase
APAP	Acetaminophen (N-acetyl-para-aminophenol)
AST	Aspartate aminotransferase
Bax	Bcl-2-associated X
Bcl2	B-cell lymphoma 2
Caspase	Cysteinyl aspartate specific proteinase
CYP2E1	Cytochrome P4502E1
GSH	Glutathione
HO-1	Heme oxygenase-1
HPLC	High-performance liquid chromatography
MDA	Malondialdehyde
MPO	Myeloperoxidase
NAPQI	N-acetyl-p-benzoquinone imine
NF-κB	Transcription factors nuclear factor-kappa B
Nrf2	Nuclear factor erythroid 2-related factor 2
qPCR	quantitative polymerase chain reaction
ROS	Reactive oxygen species
SOD	Superoxide dismutase
TAE	Triticum aestivum sprouts extract
UPLC	Ultra-performance liquid chromatography
